# Relationship of common variants in *VEGFA* gene with osteonecrosis of the femoral head: A Han Chinese population based association study

**DOI:** 10.1038/s41598-018-34352-4

**Published:** 2018-11-01

**Authors:** Wenlong Ma, Kun Xin, Ke Chen, Hongtao Tang, Honggan Chen, Liqiang Zhi, Hongliang Liu

**Affiliations:** 1 Department of Hip Injury and Disease, Luoyang Orthopedic Hospital of Henan Province, Luoyang, Henan China; 2 Department of orthopedics, Taihe Hospital of Traditional Chinese Medicine, Taihe, Anhui China; 3 Department of Joint Surgery, Honghui Hospital, Xi’an Jiaotong University, Xi’an, Shaanxi China; 4 Department of Trauma, Honghui Hospital, Xi’an Jiaotong University, Xi’an, Shaanxi China

## Abstract

The pathology of non-traumatic osteonecrosis of the femoral head (ONFH) is complex. Several studies have linked some polymorphisms of vascular endothelial growth factors A (*VEGFA*) with ONFH, but the results are not consistent and are even conflicting. In the study, 22 single nucleotide polymorphisms (SNPs) in *VEGFA* were genotyped in 1,762 subjects (489 cases and 1,273 controls). Genetic association analyses were performed in single markers and haplotype levels. Stratification analysis was conducted for ONFH patients. Gene by environment interactions were tested between *VEGFA* and the smoking status of the subjects. Gene expression and eQTL data of significant SNPs were extracted from GTEx to examine their potential biological function. The SNP, rs2010963, was identified to be significantly associated with ONFH (χ^2^ = 11.66, *P* = 0.0006, OR = 1.29). Haplotypes including rs2010963 were also identified to be correlated with ONFH in the haplotype-based analyses. After stratifying by the causes of ONFH, a significant signal from rs2010963 could only be identified in alcohol-induced patients (*P*_allelic_ = 0.0009) but not in steroid-induced patients (*P*_allelic_ = 0.055). No significant results were obtained from the gene by environmental interaction analyses. Significant expression differences of *VEGFA* were identified in multiple human tissues for different genotypes of rs2010963. Our findings indicate that SNP rs2010963 is significantly associated with ONFH.

## Introduction

Osteonecrosis of the femoral head (ONFH) is a complex bone disorder characterized by the death of bone cells due to insufficient blood flow. The death of bone cells can, in turn, lead to pain and collapse of particular areas of bone^1,2^. Several causes of this disorder have been identified through clinical and epidemiology studies, and the two most important causes for non-traumatic ONFH are high-dose corticosteroid medications and excessive alcohol consumption^1^. The pathology of non-traumatic ONFH is complex, and multiple metabolic pathways are involved^3^. Recently, candidate gene-based association studies have successfully mapped susceptibility for many complex diseases^4–10^. Investigations of the genetic etiology of ONFH will enable us to unravel the biological and physiological mechanisms of ONFH and will also provide a basis for the development of personalized treatment for this disorder.

Gene vascular endothelial growth factor A (*VEGFA*) is located on chromosome 6p31.3. It encodes a member of the vascular endothelial growth factors (VEGFs)^11^. Several previous studies have linked multiple genetic polymorphisms within the promoter region of *VEGFA* to the disease status of non-traumatic ONFH^12–14^. Despite the significant findings that have been reported, most of these studies had poor coverage of genetic markers on *VEGFA* and low statistical power due to small sample sizes. In addition, the results of these previous studies are not consistent and are even conflicting in some cases. Several different variants located in the promoter region of *VEGFA* have been shown to contribute to the risk of ONFH; however, the roles of these SNPs are still unclear. It is difficult to tell whether these SNPs are surrogates of some underlying susceptible variants or genetic markers with biologically functional significance.

In this study, we aimed to investigate the genetic association between *VEGFA* and ONFH in a large Chinese study sample. Through genotyping several pre-selected genetic markers covering *VEGFA* in our study subjects, we examined the statistical association between genetic polymorphisms and ONFH in both single-marker and haplotype-based methods. In addition, combined with relevant bioinformatics tools, we aimed to examine the potential biological function of the significant SNPs identified in the association analysis.

## Methods

### Study Subjects

In the study, a total of 489 unrelated male patients with non-traumatic ONFH and 1,273 unrelated control subjects were consecutively recruited at the Luoyang Orthopedic Hospital of Henan Province (Luoyang, China) from 2013 to 2016. Patients were diagnosed according to assessment by X-rays, magnetic resonance imaging (MRI), and bone scans. Based on the etiological factors of ONFH, patients were divided into a steroid-induced group (254 cases) and an alcohol-induced group (235 cases). Steroid-induced ONFH was defined by a history of a mean daily dose of ≥16.6 mg or a highest daily dose of 80 mg of prednisolone equivalent within 1 year before the development of symptoms or radiological diagnosis in asymptomatic cases. When steroid and other factors were excluded, patients with a history of ethanol consumption of at least 400 ml per week for at least 1 year were categorized under alcohol-induced ONFH. Patients with a demonstrable history of direct trauma or with possible combined causes were excluded. Those who had a chronic metabolic disorder of the heart, kidney, or liver were also excluded. Control subjects were matched with patients for age and BMI and were enrolled from subjects attending routine medical checkups. The controls had no hip pain, and anteroposterior and frog-leg lateral pelvic radiographs did not show any lesions. The controls had a history of ethanol consumption of at least 400 ml per week for at least 1 year; however, they had no alcohol-induced ONFH or other related diseases, no history of thromboembolic events and no symptoms of hip disease. All participants were restricted to the Han Chinese population who lived in Luoyang city and surrounding areas. Informed consent was obtained from all groups. The study protocol conformed to the ethical guidelines of the 1975 Declaration of Helsinki and was approved by the Ethics Committee of Luoyang Orthopedic Hospital of Henan Province.

### SNP Selection and Genotyping

We searched for all SNPs with minor allele frequencies (MAF) ≥ 0.05 within the region of the *VEGFA* gene in the 1000 Genomes Chinese Han Beijing population (CHB). Then, MAF ≥ 0.05 with pair-wise tagging and *r*^2^ ≥ 0.8 were used as the cut-off criteria during tag SNP selection, which generated 22 tag SNPs covering the region of the *VEGFA* gene for our study. Basic information on the 22 selected SNPs is summarized in Supplemental Table S1. Genomic DNA was isolated from peripheral blood using a Tiangen DNA extraction kit (Tiangen Biotech Co. Ltd, Beijing, China) according to the manufacturer’s protocol. SNP genotyping was performed using a Sequenom MassARRAY platform with iPLEX GOLD chemistry (Sequenom, San Diego, CA, USA) based on the manufacturer’s protocols. The results were processed using Sequenom Typer 4.0 software, and genotype data were generated from the samples^15^. Genotyping was conducted by laboratory personnel blinded to the case-control status, and the genotyping results, data entry and statistical analyses were independently reviewed by two authors. We randomly re-performed the analysis on 5% of the sample, with a concordance of 100%.

### Statistical and Bioinformatic Methods

χ^2^ tests were performed using Plink^16^ for each marker to examine the potential association between SNPs and ONFH disease status. Genomic control (GC) was conducted to identify and correct potential false positive signals due to population stratification. Linkage disequilibrium (LD) blocks were constructed for the 22 selected SNPs, and haplotype-based association tests were performed using Haploview^17^. Significant SNPs identified by single-marker-based tests were re-analyzed in stratification analysis. In this analysis, our patients were stratified by the clinical type of ONFH (alcohol-induced or steroid-induced). To investigate the potential gene by environment interactions between selected SNPs of *VEGFA* and the smoking status of our study subjects, we performed G-by-E interaction analysis by fitting logistic models with a multiplying term. R project^18^ was utilized for general statistical computing and G-by-E interaction analysis. Bonferroni corrections were applied to address multiple comparisons. For single-marker-based analysis, our *P* value threshold was 0.05/22 ≈ 0.002.

RegulomeDB was utilized to examine the potential biological functions of selected SNPs^19^. We investigated the potential effects of significant SNPs on the gene expression of *VEGFA* using the database of GETx (https://www.gtexportal.org/home/)^20^. Data for gene expression of *VEGFA* in 47 human tissues were extracted and compared among different genotypes of significant SNPs identified in association tests.

## Results

### Genetic association between polymorphisms of VEGFA and ONFH

The clinical characteristics of all subjects are presented in Table 1. There were no differences in age, body mass index (BMI) or smoking status between the patients and controls. All 22 selected SNPs (Supplemental Table S1) passed the Hardy-Weinberg Equilibrium (HWE) test. We identified one significant SNP, rs2010963 (χ^2^ = 11.66, *P* = 0.0006, OR = 1.29, RegulomeDB Score = 4), as being associated with the disease status of ONFH (Table 2). The C allele of rs2010963 is related to a higher risk of ONFH. Two other SNPs showed nominal significance but failed to persist after multiple comparison corrections. The median of the χ^2^ statistics for our selected SNPs was 0.15, which is far smaller than the expected value of 0.456. Therefore, no significant population stratification could be detected in our data (the Q-Q plot is shown in Supplemental Fig. S1).

**Table 1:** The clinical characteristics of the subjects.

Characteristics	Subjects (N = 1,762)		*P*-value
	Patients (N = 489)	Controls (N = 1,273)	
Age (years), mean ± SD	50.5 ± 7.4	50.5 ± 8.5	0.8844
BMI (kg/m^2^), mean ± SD	24.8 ± 0.96	24.7 ± 0.99	0.1136
Smoking status (yes/no)	192/653	394/1357	0.9512
Classified by causes(%)			
Alcohol-induced	235 (48)	—	—
Steroid-induced	254 (52)	—	—

SD: standard deviation; BMI, body mass index. Student t tests were performed for Age and BMI between patients and controls group. χ^2^ was conducted for distribution of smoking status and patients and controls group.

**Table 2:** Results of single marker based genetic association tests for 22 selected SNPs.

CHR	SNP	BP	A1	F_A	F_U	CHISQ	*P*	OR	RegSc
6	rs699947	43768652	A	0.21	0.25	5.13	0.0235	0.81	5
6	rs1570360	43770093	A	0.14	0.18	6.24	0.0125	0.77	4
**6**	**rs2010963**	**43770613**	**C**	**0.5**	**0.43**	**11.66**	**0.0006**	**1.29**	**4**
6	rs25648	43771240	T	0.1	0.1	0	0.9635	0.99	4
6	rs113747229	43771590	T	0.08	0.08	0.16	0.691	1.06	4
6	rs1885657	43772357	C	0.29	0.29	0.11	0.7448	1.03	4
6	rs3024987	43773103	T	0.17	0.17	0.04	0.8412	0.98	4
6	rs62401162	43779212	C	0.13	0.12	0.3	0.5817	1.07	4
6	rs3025006	43779511	C	0.42	0.42	0.13	0.7229	1.03	3a
6	rs3025007	43779634	T	0.26	0.27	0.14	0.7065	0.97	4
6	rs3025010	43779840	C	0.24	0.23	0.56	0.4547	1.07	4
6	rs3025011	43779886	T	0.09	0.1	0.39	0.5323	0.92	2b
6	rs3025012	43780225	G	0.14	0.15	0.12	0.7253	0.96	4
6	rs3025017	43780620	A	0.12	0.11	0.04	0.8393	1.02	4
6	rs3025020	43781373	T	0.35	0.33	0.67	0.4124	1.07	4
6	rs3025021	43781426	T	0.19	0.2	0.15	0.7015	0.96	2b
6	rs3025029	43782819	A	0.18	0.18	0.05	0.8244	0.98	4
6	rs3025032	43783300	T	0.1	0.1	0.15	0.7022	0.95	4
6	rs3025035	43783622	T	0.17	0.17	0.21	0.6447	1.05	3a
6	rs3025036	43783932	G	0.14	0.14	0.15	0.6947	1.04	3a
6	rs10434	43785475	A	0.21	0.21	0.07	0.7881	1.03	4
6	rs3025053	43785588	A	0.09	0.09	0.05	0.8306	0.97	4

CHR: chromosome; POS: position; A1: tested allele; F_A: allele frequency of tested allele in affected subjects; F_U: allele frequency of tested allele in unaffected subjects; HWE: *P* values of Hardy-Weinberg equilibrium tests conducted for unaffected subjects; CHISQ: χ^2^ statistics; RegSc: RegulomeDB score. Significant results were highlighted in bold.

**Figure 1:**
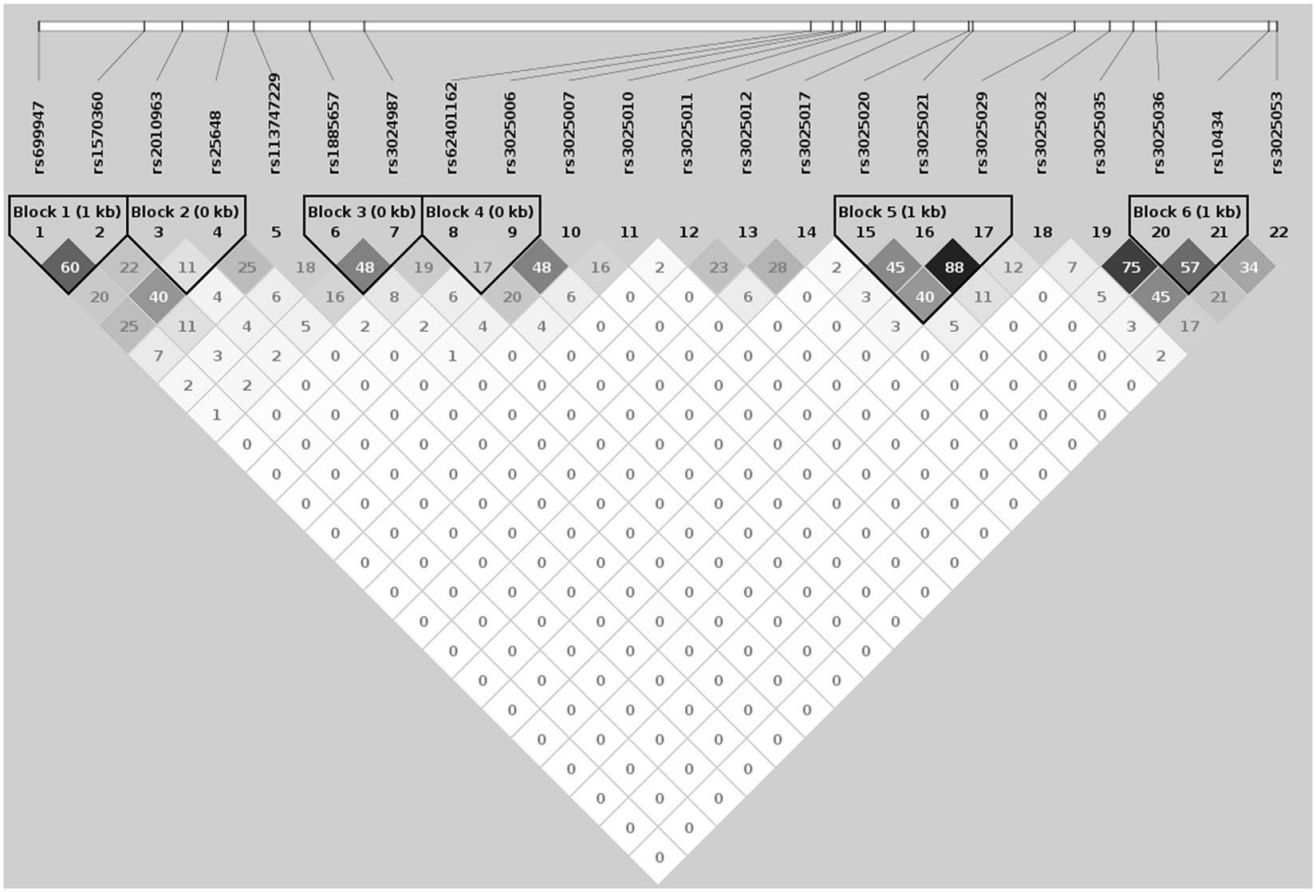
LD structure of the 22 selected SNPs. Values of *r*^2^ were indicated in each square and were used as color scheme for this plot.

Six LD blocks were constructed based on our data (Fig. 1). The *P* value threshold used here was 0.05/6 = 0.008. One significant LD block was identified as being associated with the disease status of ONFH (χ^2^ = 11.66, *P* = 0.0007). This LD block included two SNPs, rs2010963-rs25648, covering a region of 630 base pairs in *VEGFA* (Table 3). Another LD block, rs699947-rs1570360, showed nominal significance (*P* = 0.023).

**Table 3:** Results of haplotype based association analysis.

LOCUS	HAPLOTYPE	F_A	F_U	χ^2^	DF	*P*	SNPs
H1	OMNIBUS	-	-	7.55	2	0.0230	rs699947|rs1570360
H1	AA	0.14	0.18	7.54	1	0.0060	rs699947|rs1570360
H1	AG	0.08	0.07	0.09	1	0.7687	rs699947|rs1570360
H1	CG	0.79	0.75	4.89	1	0.0270	rs699947|rs1570360
**H2**	**OMNIBUS**	**—**	**—**	**14.48**	**2**	**0.0007**	**rs2010963|rs25648**
H2	CT	0.09	0.09	0.05	1	0.8225	rs2010963|rs25648
**H2**	**CC**	**0.41**	**0.34**	**13.99**	**1**	**0.0002**	**rs2010963|rs25648**
**H2**	**GC**	**0.50**	**0.57**	**12.06**	**1**	**0.0005**	**rs2010963|rs25648**
H3	OMNIBUS	**—**	**—**	1.22	2	0.5427	rs1885657|rs3024987
H3	CT	0.17	0.17	0.20	1	0.6546	rs1885657|rs3024987
H3	CC	0.13	0.12	1.15	1	0.2839	rs1885657|rs3024987
H3	TC	0.70	0.71	0.16	1	0.6913	rs1885657|rs3024987
H4	OMNIBUS	**—**	**—**	0.21	2	0.9015	rs62401162|rs3025006
H4	CC	0.12	0.12	0.11	1	0.7449	rs62401162|rs3025006
H4	TC	0.30	0.30	0.05	1	0.8173	rs62401162|rs3025006
H4	TT	0.57	0.58	0.18	1	0.6689	rs62401162|rs3025006
H5	OMNIBUS	**—**	**—**	3.57	3	0.3112	rs3025020|rs3025021|rs3025029
H5	TTA	0.17	0.18	0.42	1	0.5161	rs3025020|rs3025021|rs3025029
H5	TTG	0.02	0.02	0.09	1	0.7583	rs3025020|rs3025021|rs3025029
H5	TCG	0.16	0.14	3.34	1	0.0678	rs3025020|rs3025021|rs3025029
H5	CCG	0.65	0.67	0.83	1	0.3623	rs3025020|rs3025021|rs3025029
H6	OMNIBUS	**—**	**—**	0.15	2	0.9270	rs3025036|rs10434
H6	GA	0.14	0.14	0.01	1	0.9056	rs3025036|rs10434
H6	CA	0.08	0.07	0.13	1	0.7223	rs3025036|rs10434
H6	CG	0.79	0.79	0.11	1	0.7440	rs3025036|rs10434

DF: degree of freedom. Significant LD block was highlighted in bold. F_A: frequency of haplotypes in cases. F_U: frequency of haplotypes in controls.

Stratification analyses for rs2010963 were performed at both the genotypic and allelic level (Table 4). Interestingly, after we stratified patients by their clinical type, significant signal from rs2010963 was only identified in alcohol-induced patients (*P*_allelic_ = 0.0009) but not in steroid-induced patients (*P*_allelic_ = 0.055). This discordance was identified in both genotypic and allelic analysis.

**Table 4:** Stratification analysis of genetic association of SNP rs2010963 for different clinical type of osteonecrosis.

	Genotypic Analysis			χ^2^	*P*	Allelic Analysis		χ^2^	*P*
	CC (N = 517)	CG (N = 898)	GG (N = 347)			C (N = 1932)	G (N = 1592)		
Controls	401	639	233			1441	1105		
Alcohol_induced patients	49	129	57	12.05	0.0024	227	243	11.06	0.0009
Steroid_induced patients	67	130	57	3.76	0.1524	264	244	3.68	0.0550

No significant results were obtained through gene-by-environment-interaction analyses. The most significant signal identified was from rs3025020 (*P* = 0.0184, OR = 0.69). However, it failed to persist following Bonferroni correction (Supplemental Table S2).

### Effects of significant SNPs on gene expression of VEGFA

**Figure 2:**
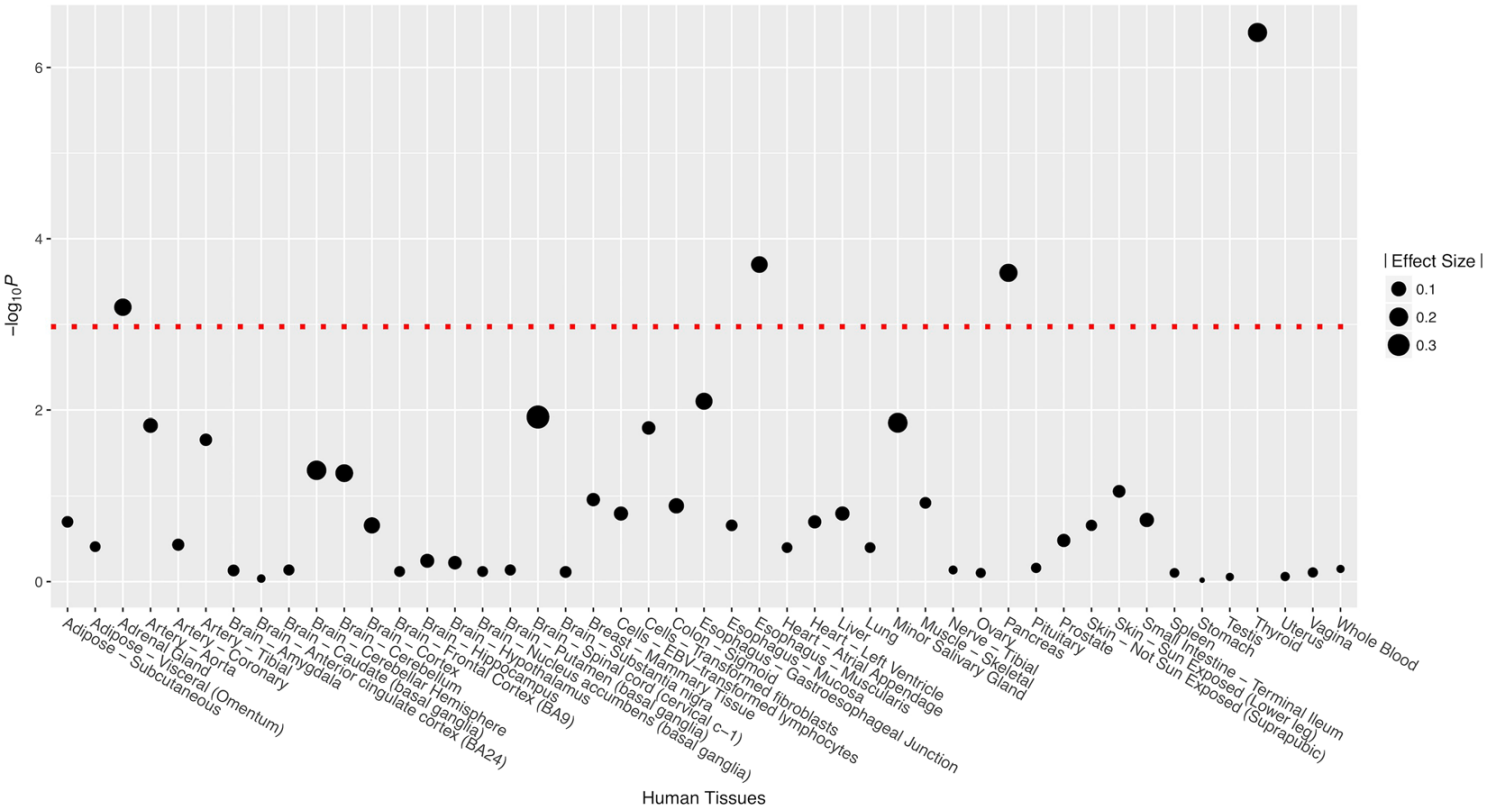
Effects of rs2010963 on gene expression of VEGF in 47 different human tissues. *P* value threshold was indicated by red dotted line.

We investigated the effects of SNP rs2010963 on the expression of *VEGFA* by examining the eQTL data from 47 normal human tissues extracted from GTEx (Supplemental Table S3). Significant expression differences were identified in four human tissues: the adrenal gland, esophagus muscularis, pancreas and thyroid (Fig. 2). The most significant difference was in the thyroid, with *P* < 1 × 10^−6^. The C allele of rs2010963 was related to higher expression of *VEGFA*. These findings indicate that rs2010963 is an eQTL for *VEGFA*. In addition, SNPs capturing eQTL signals with genome-wide significance were summarized in Supplemental Table S4. As shown, six out of these 22 selected SNPs had eQTL signals with genome-wide significance.

## Discussion

*VEGFA* encodes a heparin-binding protein that is an important member of the VEGF growth factor family^21–25^. Earlier studies have shown that this growth factor promotes the proliferation and migration of vascular endothelial cells and plays an important role in the physiology of angiogenesis. Knock-out of this gene in mice results in abnormal blood vessel formation in the embryonic stage. In addition to its role in angiogenesis, this growth factor has also been shown to be essential for the formation of endochondral bone. Gerber *et al*. investigated the potential role of *VEGFA* in the formation of endochondral bone by inactivating VEGF in 24-day-old mice^26^. The results showed that proliferation, differentiation and maturation of chondrocytes were basically normal, but resorption of terminal chondrocytes was significantly inhibited. These findings indicate that VEGF is an essential coordinator of bone formation in the growth plate^26^.

Compared to previous studies based on Asian populations, which resulted in inconsistent findings about the significant SNPs of *VEGFA*^12,13^, in this study, we identified rs2010963 (−634C/G or +450 C/G as indicated in some other studies) to be associated with the disease status of ONFH. Some SNPs, such as rs699947 (−2578A/C) and rs1570360 (−1154A/G), which were shown to be significantly associated with this bone disorder in other studies, were shown to be only surrogates of rs2010963 in our study. These SNPs had nominal significance with ONFH in our data only because they are in medium LD with rs2010963. Given that limited SNPs analyses were difficult to draw reliable and stable conclusions^27–31^,our further haplotype-based analyses provided more evidence about the role of rs2010963 and its surrounding genomic regions in the susceptibility to ONFH. In our study, the C allele would increase the risk of ONFH by approximately 20% compared to the G allele. The eQTL analyses showed that rs2010963 is a potential eQTL of *VEGFA* and can affect the gene expression of *VEGFA* in multiple normal human tissues. The C allele of rs2010963 is related to higher expression of *VEGFA*. Combined with the results from gene expression analysis, we believe that SNP rs2010963 (−634C/G) might be more than just a surrogate of some underlying ungenotyped genetic variants but a variant with specific biologically functional significance. However, because this was a genetic association study, it is impossible for us to unravel the potential link between increased expression of *VEGFA* and disease risk of ONFH from this study alone, and more research is needed in the future.

One interesting result of this study is that by stratifying our ONFH patients, we found that a significant signal for rs2010963 could only be identified among alcohol-induced ONFH patients and not among steroid-induced patients. Because the alcohol-induced group had a smaller sample size compared to the steroid-induced group, this discordance cannot be explained by decreased statistical power due to a smaller sample size caused by stratification. One potential explanation is that this difference might be caused by some undetected selection bias in our patient recruitment process. On the other hand, an alternative explanation, which might be more informative, is that this difference might indicate some deeper difference in the biological mechanisms for the two types of ONFH patients. Still, more studies with larger sample sizes are needed in the future to replicate and validate these findings.

This study has several limitations. Firstly, in this study, we only recruited males as the study subjects, and this may impair the generalization of the study results. Most previous similar studies enrolled both males and females, although the ratios of the genders are always imbalanced^12–14^. Secondly, due to the limitations of our study design, although we extracted gene expression data from GTEx for normal human tissues, it might be more meaningful to quantify the gene expression of *VEGFA* and estimate the differences of gene expression between ONFH patients and controls in our own study subjects. In addition, another potential limitation is that we only included common polymorphisms (SNPs with MAF > 0.05). Low-frequency and rare variants were not included in this study. In this sense, the present study is incomplete and is unable to systematically unravel the potential genetic architecture for osteonecrosis, and our results should be considered to be preliminary and confirmed in the future research. Thirdly, we did not control the exposure to steroids in our control samples and this would be a potential flaw in study design. This flaw might be, at least partly, responsible for the non-significant findings in stratification analysis for steroid-induced patients, but the main significant results in the study would not be affected by the limitation.

In summary, our study systematically examined the genetic association between SNPs in the *VEGFA* gene based on study subjects of Chinese ethnic groups. Our findings indicate that SNP rs2010963 is significantly associated with the risk of ONFH, which provide additional supportive evidence of the relationship between *VEGFA* gene and ONFH. Comprehensive investigation with more SNPs, different populations, larger sample sizes and functional experiments are prospected to validate our results, understand the effects of VEGFA on the risk of ONFH, and elucidate the potential biological mechanisms of ONFH.

## Electronic supplementary material

Supplemental Materials
