# Supplementary material for: Relationship of common variants in VEGFA gene with osteonecrosis of the femoral head: A Han Chinese population based association study
Source: Sci Rep. 2018 Nov 1;8:16221. doi: 10.1038/s41598-018-34352-4 (PMC6212426; doi:10.1038/s41598-018-34352-4)
Supplement: Supplementary file 1 — Supplemental Materials [file 41598_2018_34352_MOESM1_ESM.docx]

***Title***: Relationship of common variants in *VEGFA* gene with osteonecrosis of the femoral head: A Han Chinese population based association study

***Author names and affiliations***: Wenlong Ma ^1*^, Kun Xin ^2*^, Ke Chen ^1^, Hongtao Tang ^1^, Honggan Chen ^1^, Liqiang Zhi ^3^ and Hongliang Liu ^4^

^1^ Department of Hip Injury and Disease, Luoyang Orthopedic Hospital of Henan Province, Luoyang, Henan, China;

^2^ Department of orthopedics, Taihe Hospital of Traditional Chinese Medicine, Taihe, Anhui, China;

^3^ Department of Joint Surgery, Honghui Hospital，Xi’an Jiaotong University, Xi'an, Shaanxi, China;

^4^ Department of Trauma, Honghui Hospital，Xi’an Jiaotong University, Xi'an, Shaanxi, China.

*These authors contributed equally to the work.

***Corresponding Author***:

Liqiang Zhi, M.D. & Ph.D., Department of Joint Surgery, Honghui Hospital, Xi’an Jiaotong University Health Science Center, No.555, Youyi East Road, Xi'an, Shaanxi, China, 710054.

Tel: 86-29-88418009; Fax: 86-29-62818386; E-mail: osteolqzhi@163.com

Hongliang Liu, M.D. & Ph.D., Department of Trauma, Honghui Hospital, Xi’an Jiaotong University Health Science Center, No.555, Youyi East Road, Xi'an, Shaanxi, China, 710054.

Tel: 86-29-88418023; Fax: 86-29-62818386; E-mail: osteohlliu@163.com

Supplemental Table S1. Basic information for the 22 selected SNPs.

| CHR | POS | SNP | Alleles | avHet | FUNC |
| --- | --- | --- | --- | --- | --- |
| 6 | 43785475 | rs10434 | A/G | 0.45 | untranslated-3 |
| 6 | 43771590 | rs113747229 | C/T | 0.07 | intron,near-gene-5 |
| 6 | 43770093 | rs1570360 | A/G | 0.31 | near-gene-5 |
| 6 | 43772357 | rs1885657 | C/T | 0.44 | intron |
| 6 | 43770613 | rs2010963 | C/G | 0.44 | near-gene-5,untranslated-5 |
| 6 | 43771240 | rs25648 | C/T | 0.27 | coding-synon,near-gene-5,untranslated-5 |
| 6 | 43773103 | rs3024987 | C/T | 0.29 | intron |
| 6 | 43779511 | rs3025006 | C/T | 0.49 | intron |
| 6 | 43779634 | rs3025007 | C/T | 0.48 | intron |
| 6 | 43779840 | rs3025010 | C/T | 0.46 | intron |
| 6 | 43779886 | rs3025011 | A/T | 0.11 | intron |
| 6 | 43780225 | rs3025012 | A/G | 0.22 | intron |
| 6 | 43780620 | rs3025017 | A/G | 0.11 | intron |
| 6 | 43781373 | rs3025020 | C/T | 0.36 | intron |
| 6 | 43781426 | rs3025021 | C/T | 0.39 | intron |
| 6 | 43782819 | rs3025029 | A/G | 0.24 | intron |
| 6 | 43783300 | rs3025032 | C/T | 0.34 | intron |
| 6 | 43783622 | rs3025035 | C/T | 0.27 | intron |
| 6 | 43783932 | rs3025036 | C/G | 0.35 | intron |
| 6 | 43785588 | rs3025053 | A/G | 0.16 | untranslated-3 |
| 6 | 43779212 | rs62401162 | C/T | 0.23 | intron |
| 6 | 43768652 | rs699947 | A/C | 0.44 | near-gene-5 |

CHR: chromosome; POS: genomic position; avHet: average of heterogosity; FUNC: function.

Supplemental Table S2. Results of the gene by environmental factor analysis for 22 selected SNPs.

| SNP | ENV | Z statistics | *P* | OR |
| --- | --- | --- | --- | --- |
| rs699947_A | smoking | 0.72 | 0.4686 | 1.14 |
| rs1570360_A | smoking | 0.64 | 0.5205 | 1.15 |
| rs2010963_C | smoking | 0.30 | 0.7679 | 1.05 |
| rs25648_T | smoking | -0.70 | 0.4813 | 0.84 |
| rs113747229_T | smoking | 0.25 | 0.8046 | 1.07 |
| rs1885657_C | smoking | -0.61 | 0.5432 | 0.90 |
| rs3024987_T | smoking | -0.65 | 0.5188 | 0.88 |
| rs62401162_C | smoking | -0.27 | 0.7872 | 0.94 |
| rs3025006_C | smoking | 0.32 | 0.7468 | 1.05 |
| rs3025007_T | smoking | -1.07 | 0.2835 | 0.83 |
| rs3025010_C | smoking | 1.01 | 0.3122 | 1.20 |
| rs3025011_T | smoking | -0.69 | 0.4886 | 0.84 |
| rs3025012_G | smoking | -0.02 | 0.9866 | 1.00 |
| rs3025017_A | smoking | -0.72 | 0.4685 | 0.84 |
| rs3025020_T | smoking | -2.36 | 0.0184 | 0.69 |
| rs3025021_T | smoking | -1.56 | 0.1197 | 0.74 |
| rs3025029_A | smoking | -0.99 | 0.3228 | 0.82 |
| rs3025032_T | smoking | 0.74 | 0.4572 | 1.21 |
| rs3025035_T | smoking | 0.79 | 0.4316 | 1.17 |
| rs3025036_G | smoking | 0.45 | 0.6557 | 1.10 |
| rs10434_A | smoking | 0.35 | 0.7229 | 1.07 |
| rs3025053_A | smoking | -0.11 | 0.9135 | 0.97 |

ENV: environmental factor.

Supplemental Table S3. eQTL data for rs2010963 on gene *VEGFA*.

| Gene Symbol | SNP | *P* | Effect Size | T-Statistic | Tissue |
| --- | --- | --- | --- | --- | --- |
| *VEGFA* | rs2010963 | 3.90×10^-7^ | -0.21 | -5.2 | Thyroid |
| *VEGFA* | rs2010963 | 0.0002 | -0.14 | -3.8 | Esophagus - Muscularis |
| *VEGFA* | rs2010963 | 0.00025 | -0.18 | -3.7 | Pancreas |
| *VEGFA* | rs2010963 | 0.00063 | -0.16 | -3.5 | Adrenal Gland |
| *VEGFA* | rs2010963 | 0.0079 | -0.15 | -2.7 | Esophagus - Gastroesophageal Junction |
| *VEGFA* | rs2010963 | 0.012 | -0.33 | -2.6 | Brain - Spinal cord (cervical c-1) |
| *VEGFA* | rs2010963 | 0.014 | -0.22 | -2.5 | Minor Salivary Gland |
| *VEGFA* | rs2010963 | 0.015 | -0.096 | -2.5 | Artery - Aorta |
| *VEGFA* | rs2010963 | 0.016 | -0.073 | -2.4 | Cells - Transformed fibroblasts |
| *VEGFA* | rs2010963 | 0.022 | -0.056 | -2.3 | Artery - Tibial |
| *VEGFA* | rs2010963 | 0.05 | -0.22 | -2 | Brain - Cerebellar Hemisphere |
| *VEGFA* | rs2010963 | 0.054 | -0.17 | -1.9 | Brain - Cerebellum |
| *VEGFA* | rs2010963 | 0.088 | -0.06 | -1.7 | Skin - Sun Exposed (Lower leg) |
| *VEGFA* | rs2010963 | 0.11 | 0.071 | 1.6 | Breast - Mammary Tissue |
| *VEGFA* | rs2010963 | 0.12 | -0.044 | -1.6 | Muscle - Skeletal |
| *VEGFA* | rs2010963 | 0.13 | -0.11 | -1.5 | Colon - Sigmoid |
| *VEGFA* | rs2010963 | 0.16 | -0.086 | -1.4 | Cells - EBV-transformed lymphocytes |
| *VEGFA* | rs2010963 | 0.16 | -0.088 | -1.4 | Liver |
| *VEGFA* | rs2010963 | 0.19 | -0.089 | -1.3 | Small Intestine - Terminal Ileum |
| *VEGFA* | rs2010963 | 0.2 | 0.043 | 1.3 | Adipose - Subcutaneous |
| *VEGFA* | rs2010963 | 0.2 | -0.068 | -1.3 | Heart - Left Ventricle |
| *VEGFA* | rs2010963 | 0.22 | -0.13 | -1.2 | Brain - Cortex |
| *VEGFA* | rs2010963 | 0.22 | -0.044 | -1.2 | Esophagus - Mucosa |
| *VEGFA* | rs2010963 | 0.22 | -0.039 | -1.2 | Skin - Not Sun Exposed (Suprapubic) |
| *VEGFA* | rs2010963 | 0.33 | -0.069 | -0.97 | Prostate |
| *VEGFA* | rs2010963 | 0.37 | -0.051 | -0.9 | Artery - Coronary |
| *VEGFA* | rs2010963 | 0.39 | -0.033 | -0.86 | Adipose - Visceral (Omentum) |
| *VEGFA* | rs2010963 | 0.4 | 0.032 | 0.84 | Heart - Atrial Appendage |
| *VEGFA* | rs2010963 | 0.4 | -0.033 | -0.85 | Lung |
| *VEGFA* | rs2010963 | 0.57 | -0.082 | -0.58 | Brain - Hippocampus |
| *VEGFA* | rs2010963 | 0.6 | -0.074 | -0.52 | Brain - Hypothalamus |
| *VEGFA* | rs2010963 | 0.69 | -0.027 | -0.4 | Pituitary |
| *VEGFA* | rs2010963 | 0.71 | -0.0096 | -0.37 | Whole Blood |
| *VEGFA* | rs2010963 | 0.73 | 0.036 | 0.35 | Brain - Caudate (basal ganglia) |
| *VEGFA* | rs2010963 | 0.73 | -0.037 | -0.34 | Brain - Putamen (basal ganglia) |
| *VEGFA* | rs2010963 | 0.73 | -0.015 | -0.34 | Nerve - Tibial |
| *VEGFA* | rs2010963 | 0.74 | 0.046 | 0.33 | Brain - Amygdala |
| *VEGFA* | rs2010963 | 0.76 | 0.034 | 0.31 | Brain - Frontal Cortex (BA9) |
| *VEGFA* | rs2010963 | 0.76 | 0.034 | 0.31 | Brain - Nucleus accumbens (basal ganglia) |
| *VEGFA* | rs2010963 | 0.77 | 0.047 | 0.3 | Brain - Substantia nigra |
| *VEGFA* | rs2010963 | 0.78 | 0.027 | 0.28 | Vagina |
| *VEGFA* | rs2010963 | 0.79 | 0.025 | 0.26 | Ovary |
| *VEGFA* | rs2010963 | 0.79 | 0.022 | 0.26 | Spleen |
| *VEGFA* | rs2010963 | 0.87 | 0.018 | 0.16 | Uterus |
| *VEGFA* | rs2010963 | 0.88 | 0.01 | 0.16 | Testis |
| *VEGFA* | rs2010963 | 0.92 | -0.012 | -0.1 | Brain - Anterior cingulate cortex (BA24) |
| *VEGFA* | rs2010963 | 0.96 | -0.0018 | -0.047 | Stomach |

Supplemental table S4. Summarized information of eQTL signals achieved genome-wide significance.

| Gene Symbol | SNP | *P* | Effect Size | Tissue |
| --- | --- | --- | --- | --- |
| *GNMT* | rs113747229 | 5.50×10^-6^ | -0.54 | Thyroid |
| *VEGFA* | rs2010963 | 3.90×10^-7^ | -0.21 | Thyroid |
| *MRPS18A* | rs3024987 | 3.20×10^-6^ | -0.24 | Cells - Transformed fibroblasts |
| *VEGFA* | rs3025006 | 6.50×10^-7^ | 0.19 | Thyroid |
| *TCTE1* | rs3025017 | 2.90×10^-5^ | 0.46 | Muscle - Skeletal |
| *POLH* | rs699947 | 2.00×10^-6^ | 0.19 | Whole Blood |


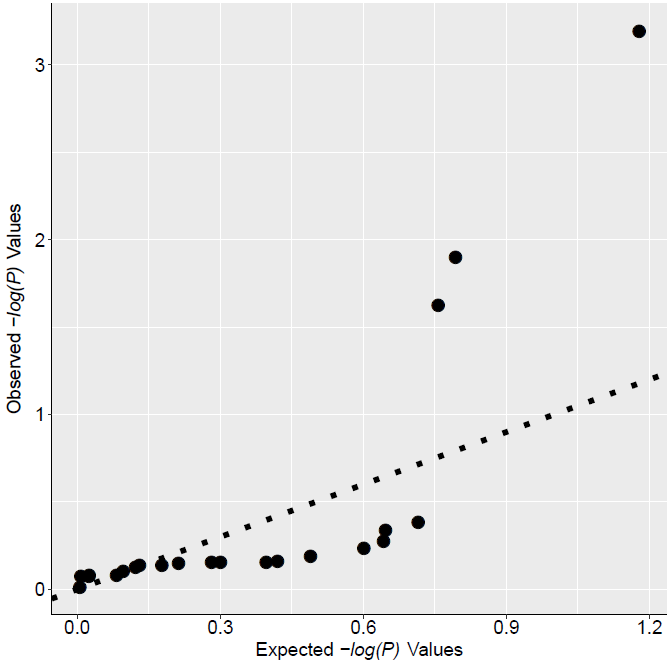


Supplemental Figure S1. Q-Q plot of the single marker based association results.
